# Tumor Endothelial Cells-Associated Integrin Alpha-6 as a Promising Biomarker for Early Detection and Prognosis of Hepatocellular Carcinoma

**DOI:** 10.3390/cancers15164156

**Published:** 2023-08-17

**Authors:** Hyung Seok Kim, Jung Hwan Yoon, Geum Ok Baek, Moon Gyeong Yoon, Ji Eun Han, Hyo Jung Cho, Soon Sun Kim, Jee-Yeong Jeong, Jae Youn Cheong, Jung Woo Eun

**Affiliations:** 1 Department of Biochemistry, College of Medicine, Kosin University, Seo-gu, Busan 49267, Republic of Korea; kimhs.onco@gmail.com (H.S.K.); jyjeong@kosin.ac.kr (J.-Y.J.); 2 Department of Pathology, College of Medicine, The Catholic University of Korea, 222 Banpo-daero, Seocho-gu, Seoul 06591, Republic of Korea; yjh0875@hanmail.net; 3 Department of Gastroenterology, Ajou University School of Medicine, 164 Worldcup-ro, Yeongtong-gu, Suwon 16499, Republic of Korea; ptok99@hanmail.net (G.O.B.); ymk8028@hanmail.net (M.G.Y.); overdream@nate.com (J.E.H.); pilgrim8107@hanmail.net (H.J.C.); cocorico99@gmail.com (S.S.K.)

**Keywords:** hepatocellular carcinoma, integrin alpha-6, prognostic marker, blood marker, tumor endothelial cells, tumor microenvironment

## Abstract

**Simple Summary:**

This study aims to validate the integrin alpha-6 (ITGA6) gene as a potential blood marker for early detection of hepatocellular carcinoma (HCC). Our methodological approach involved analyzing genomic datasets, leading to the identification of ITGA6 among differentially expressed genes in HCC. Further validation and analyses revealed consistent upregulation of ITGA6 in HCC, its predominant expression in tumor endothelial cells (TECs), and associations with pro-tumorigenic immune cells. This research highlights the potential of ITGA6 as an early HCC detection marker and its role in the tumor microenvironment, paving the way for new strategies in HCC management.

**Abstract:**

HCC remains a lethal cancer type, with early detection being critical for improved patient outcomes. This study introduces a comprehensive methodological approach to identify the ITGA6 gene as a potential blood marker for early HCC (eHCC) detection. We initially analyzed the GSE114564 dataset encompassing various stages of liver disease, identifying 972 differentially expressed genes in HCC. A refined analysis yielded 59 genes specifically differentially expressed in early HCC, including ITGA6. Subsequent validation in multiple datasets confirmed the consistent upregulation of ITGA6 in HCC. In addition, when analyzing progression-free survival (PFS) within the entire patient cohort and overall survival (OS) specifically among patients classified as tumor grade G1, the group of patients characterized by high expression levels of ITGA6 displayed an elevated risk ratio in relation to prognosis. Further analyses demonstrated the predominant expression of ITGA6 in TECs and its enrichment in angiogenesis-related pathways. Additionally, positive correlations were found between ITGA6 expression and pro-tumorigenic immune cells, but not with anti-tumorigenic immune cells. Our study elucidates the potential of ITGA6 as a blood-based marker for HCC early detection and diagnosis and its complex interplay with the tumor microenvironment. Further research may lead to novel strategies for HCC management and patient care.

## 1. Introduction

Hepatocellular carcinoma (HCC), a prevalent and highly aggressive form of liver cancer, typically presents with limited treatment options and high mortality rates [1]. Therefore, the early and precise detection of HCC is paramount to enhance patient outcomes. Recent advancements have revealed molecular markers as invaluable instruments for early detection and diagnosis of HCC. Among potential markers, ITGA6 (integrin alpha-6), and its associated tumor endothelial cells (TECs) has emerged, displaying differing expression patterns in HCC versus non-tumor liver tissues [2].

ITGA6, also recognized as CD49f, is a transmembrane glycoprotein adhesion receptor synthesized as a 140-kDa precursor. Through endoproteolytic cleavage of the C-terminal domain, this precursor is converted into two disulfide-linked polypeptides of 120 and 25 kDa [3]. ITGA6 is capable of forming heterodimers with either the β1 or β4 integrin subunits, leading to the formation of α6β1 and α6β4 integrins, respectively. These integrin complexes function as receptors for the laminin family of extracellular matrix proteins [4]. The expression patterns and functions of ITGA6 are not limited to its structural role. In fact, the overexpression of ITGA6 has been associated with multiple pathologies beyond the context of HCC. For example, the aberrant expression of ITGA6 has been linked with osteoporotic vertebral fractures in elderly women, bone metastasis and ductal carcinoma, and colon cancer-initiating cells [5,6,7]. Further, it has been implicated in the invasion, metastasis, and poor prognosis of human gallbladder carcinoma [8]. Yet the implications of ITGA6 in HCC are not confined to its expression pattern or association with other diseases. The HCC tumor microenvironment (TME) is highly immunosuppressive, necessitating the exploration of innovative treatment strategies. This is where the role of ITGA6 becomes pivotal. Overexpression of immune checkpoint molecules, such as programmed death-1 (PD-1), cytotoxic T-lymphocyte antigen 4 (CTLA-4), lymphocyte-activating gene 3 protein (LAG-3), and mucin domain molecule 3 (TIM-3), on both tumor and immune cells, paired with elevated levels of immunosuppressive cytokines, impedes T cell activity, facilitating HCC’s evasion of the immune response [9]. This relationship between ITGA6 and TECs with the immune system further accentuates the need to explore the potential of ITGA6 as an early detection marker, an area our study intends to contribute significantly.

In this study, we designed a comprehensive methodology to identify and verify ITGA6 as a viable blood marker for the early detection of HCC. To this end, our study aims to identify and verify ITGA6 as a viable blood-based marker for early detection of HCC. The procedure entailed several phases: analyzing genomic datasets, conducting serial pattern analysis via the CLICK (CLustering Identification using a Connectivity Kernel) algorithm, creating heatmap analyses of selected genes, validation using independent datasets, correlating with patient prognosis, and studying the biological functions and pathways associated with ITGA6.

The initial stage of our methodology necessitated analyzing the GSE114564 dataset, which provided a rich source of genomic data for various stages of liver diseases. A comparison between non-tumor (NT) and tumor (T) samples led to the identification of 972 differentially expressed genes in HCC. In-depth analysis revealed an interesting gene expression alteration pattern as the disease progressed. A subset of the genes from this cluster, displaying high homogeneity, was selected for further examination.

To enhance the accuracy of the gene signature, additional refinement was conducted by contrasting gene expression patterns between dysplastic nodule (DN) and early HCC (eHCC). This step resulted in identifying 59 genes specifically differentially expressed in early HCC, both against preneoplastic lesions and non-tumor liver tissues. The identified genes included CAP2 (cyclase-associated actin cytoskeleton regulatory protein 2), previously studied as a potential early stage HCC biomarker, and ITGA6, which showed an incremental increase in expression from eHCC [10,11].

We subsequently confirmed the upregulation of ITGA6 in HCC versus non-tumor tissues across multiple datasets, including GSE114564 and GSE6764 [12]. This finding remained consistent in nine additional independent HCC studies, reinforcing the diagnostic potential of ITGA6. In terms of prognostic value, our analysis using TCGA data found no significant links between ITGA6 expression and survival outcomes in the overall cohort. However, a subgroup analysis showed higher risks for poor overall survival in G1 patients with elevated ITGA6 levels.

Our analysis of single-cell RNA sequencing (scRNA-seq) data revealed the predominant expression of ITGA6 in TECs, suggesting a crucial role in the tumor microenvironment. Subsequent functional investigation of genes expressed in ITGA6-positive TECs identified enrichment in processes related to endothelial cell differentiation, cadherin binding, and angiogenesis, hinting at the involvement of ITGA6 in tumor angiogenesis and progression. Additionally, we found a positive correlation between the expression of ITGA6 and five known TEC markers. Survival analysis alongside these markers indicated that patients expressing high levels of ITGA6 and the associated markers had poorer OS and DFS outcomes.

In summary, our study underscores the potential of ITGA6 as a blood-based marker for early HCC detection and diagnosis. We provide significant insights into the molecular and clinical aspects of ITGA6 in HCC through our comprehensive analyses. The associations we observed with TECs, endothelial cell differentiation, angiogenesis-related pathways, and immune infiltration underline the complex relationship between ITGA6 and the HCC tumor microenvironment. Further investigation and validation of the diagnostic and prognostic significance of ITGA6 hold great potential for advancing HCC management and enhancing patient care. By delving deeper into the role of ITGA6 in HCC, novel strategies can be developed, offering innovative approaches for the diagnosis, prognosis, and treatment of this disease.

## 2. Materials and Methods

### 2.1. Differential Expression Analysis

In our comparative expression analysis across different patient groups, we utilized several datasets, each normalized to ensure an accurate interpretation of gene expression values. One of these was GSE114564, a major dataset collected at the Catholic University of Korea in Seoul, which has been employed in our previous study [13].

GSE114564 is a dataset of RNA-seq from tissues of different liver disease stages ranging from normal liver group to advanced HCC. The dataset consisted of 15 normal liver (NL), 20 chronic hepatitis (CH), 9 liver cirrhosis (LC), 7 dysplastic nodules (DN), 18 early HCC (eHCC), and 45 advanced HCC (aHCC). To find differentially expressed gene signatures, differential expression analysis was performed in the liver cancer group compared to the non-liver cancer group, and genes that differed by more than twofold according to Welch’s *t*-test *p* < 0.0001 were selected. Next, to find gene signatures whose expression increases as liver cancer progresses, we performed pattern analysis using the CLICK algorithm on the GSE114564 dataset. The CLICK algorithm is a clustering algorithm used in the Expander program for gene expression data. It extracts modules of strongly correlated genes to understand their biological functions. 

### 2.2. Single Cell RNA-Sequencing

Unique molecular identifier (UMI) counts and metadata of filtered cells (n = 50,023) from patients with hepatocellular carcinoma and intrahepatic cholangiocarcinoma were obtained from NCBI GEO, under accession codes GSE149614 and GSE151530. The Seurat package (v4.3.0) in R (v4.0.1), a free software environment available at http://www.r-project.org/, accessed on 5 May 2023, was used to perform log normalization, feature selection using method = ‘vst,’ scaling of data, principal component analysis (PCA), cell clustering, non-linear dimensional reduction (e.g., uniform manifold approximation and projection [UMAP] plotting), and heatmap generation. Briefly, using the FindVariableFeatures function, the most variable genes (n = 3000) across the filtered cells were selected to perform PCA on scaled data. Next, the FindNeighbors and FindClusters functions were used to construct a shared nearest neighbor (SNN) graph (‘dims = 1:30′) as well as to cluster the cells (‘resolution = 1′), respectively. To visualize cells on a 2-D UMAP plot, the RunUMAP function was used with 50 PCA dimensions (‘dims = 1:50′). Cells annotated as ‘MDSCs’ were extracted and used to generate independent Seurat objects. Except for the RunUMAP function, which uses 30 PCA dimensions (‘dims = 1:30′), the same parameters were applied for the functions mentioned above. The enrichGO function in the R clusterProfiler package (v3.18.1) was used to perform gene ontology (GO) analyses of the top 100 enriched genes. Enrichment analysis of the ‘MSigDB Hallmark 2020′ database was performed using the enrichr function in the R enrichR package (v3.0). For more detailed analysis methods, please refer to our previous study [14].

### 2.3. Patient Enrollment and Clinical Term Definitions

The blood samples and data used in this study were provided by the Biobank of Ajou University Hospital, Suwon, South Korea. The study subjects were allocated into one of four groups: healthy controls with normal liver (NL), chronic hepatitis (CH), liver cirrhosis (LC), and HCC. In this study, early-stage HCC was defined as a single lesion less than 2 cm in diameter, corresponding to modified Union for International Cancer Control (mUICC) stage I. Cohorts consisted of 32, 34, and 158 patients with CH, LC, and HCC, respectively, and 28 healthy controls. All experiments performed in this study were conducted in accordance with the ethical guidelines of the 1975 Declaration of Helsinki. The study protocol was approved by the Institutional Review Board of the Ajou University Hospital, Suwon, South Korea (AJIRB-BMR-KSP-16-365, AJIRB-BMR-SMP-17-189, AJOUIRB-KSP-2019-417, and AJOUIRB-EX-2022-389). The requirement for informed consent was waived.

### 2.4. Enzyme-Linked Immunosorbent Assay (ELISA)

This analysis consisted of both discovery and validation cohorts, each encompassing diverse patient groups. These groups included patients diagnosed with recurrent HCC (n = 57), non-recurrent HCC (n = 26), advanced HCC (avHCC, n = 26), early HCC (eHCC, n = 27), liver cirrhosis (LC, n = 33), and chronic hepatitis B (CHB, n = 34), as well as healthy control subjects (NC, n = 30). Immediately after sample collection, the serum extracted from the blood of both healthy individuals and liver disease patients was stored at −80 °C. For the assay, the samples were retrieved from storage and carefully thawed at 4 °C. Following the thawing process, we measured the concentration of the ITGA6 protein within the buffy coat of each cohort. The assay was conducted using an ELISA kit (AssayGenie, Dublin, Ireland) strictly in accordance with the manufacturer’s instructions.

### 2.5. Statistical Analysis

Data are presented as means + standard deviations (SD). The statistical significance of differences between two groups was assessed using paired Student’s *t*-test or unpaired Welch’s *t*-test using GraphPad Prism version 8.0 (GraphPad Software, San Diego, CA, USA). For multiple comparisons among three groups, one-way analysis of variance (ANOVA) with Tukey’s post hoc analysis was used. Kaplan–Meier survival curves were constructed to assess the significance of the prognostic power between two patient groups. Significant differences between survival curves were determined using the log-rank test. Receiver operating characteristic (ROC) curves were determined using IBM SPSS software (IBM SPSS Statistics for Windows, version 22.0, released 2013, IBM, Armonk, NY, USA). ROC curves were analyzed to evaluate the sensitivity, specificity, and respective areas under the ROC curve (AUROC) with 95% confidence intervals (CIs) for plasma ITGA6 protein, plasma anti-ITGA6 autoantibodies, and serum-derived EV-ITGA6. All experiments were replicated at least three times. Statistical significance was set at *p* < 0.05.

## 3. Results

### 3.1. Identifying ITGA6 as a Potential Diagnostic Biomarker for Early HCC

**Figure 1:**
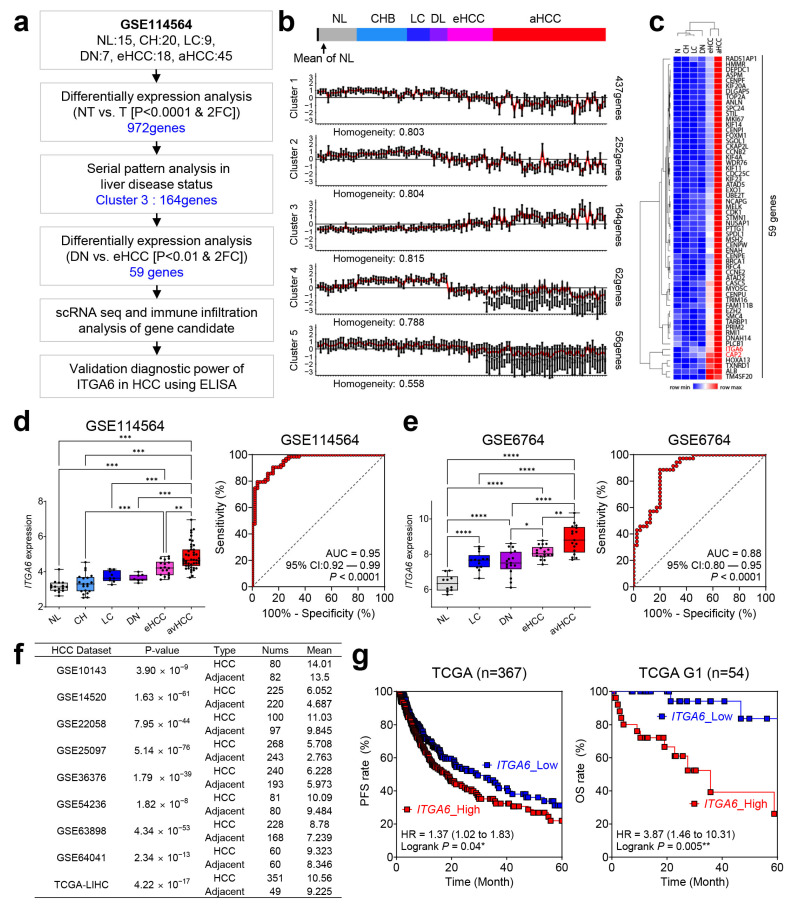
Unraveling the potential of ITGA6 in early HCC diagnosis. (**a**) Flow chart of this study’s protocol for selecting early diagnostic and immune-associated gene signatures in HCC. (**b**) Serial pattern analysis in multi-step liver disease status using CLICK algorithm. (**c**) The heatmap and hierarchical clustering analysis of differentially increased 59 genes in eHCC. (**d**) Analysis of ITGA6 expression dependent on clinical stage (left) and verification of their AUC (right) in dataset GSE114564. (**e**) Verifying ITGA6 expression by clinical stage and assessing AUC in GSE6764 dataset. (**f**) Cross-validation of ITGA6 expression in the nine additional HCC datasets. (**g**) Investigation of the correlation between ITGA6 expression and Kaplan-Meier survival expectations in liver cancer patients. NL; normal liver, CH; chronic hepatitis, LC; liver cirrhosis, DN; dysplastic nodule. * *p* < 0.05, ** *p* < 0.01, *** *p* < 0.001, **** *p* < 0.0001.

To identify early biomarkers for HCC, we utilized the GSE114564 dataset, which contains genomic data of various liver disease stages. The overall workflow of this analysis is summarized in Figure 1a.

As an initial step in this analysis, a total of 927 differentially expressed genes between NT and T samples were categorized into five clusters using the CLICK algorithm in the Expander tool [15]. Among the five clusters, Cluster three, which demonstrated the highest homogeneity score (0.815), showed an increasing expression pattern beginning at the eHCC stage and included a total of 164 genes (Figure 1b).

From these 164 genes, a selection of 59 genes, showing significant differences between the premalignant lesion DN and eHCC, was further narrowed down. Upon performing hierarchical clustering, the non-cancerous group, comprising N, CH, LC, and DN, distinctly clustered together. Notably, within these 59 genes, ITGA6 and CAP2 showed progressively increasing expression patterns starting from an early stage of HCC (Figure 1c). The aim of our study was to identify potential biomarkers for the diagnosis of early-stage liver cancer that could be detected in the blood. Given that CAP2 was already identified as a marker for early-stage liver cancer patients with negative alpha fetoprotein (AFP), we decided to focus our research analysis on the potential of ITGA6 as a diagnostic marker for early-stage liver cancer [10,11]. In line with this, our observations confirmed a relatively low expression of ITGA6 in healthy liver compared to the other tissues (Figure S1A). Further analysis of ITGA6 expression across 33 cancer types from the TCGA database revealed that ITGA6 was upregulated in tumor tissues compared to normal tissues in 13 different cancers (Figure S1B). These findings suggest that ITGA6 might play an oncogenic role in cancer development.

Upon examination of the expression of ITGA6 in the multistage liver disease dataset GSE114564, a significant increase in expression was noted, and AUC analysis also confirmed the superior performance of ITGA6 as an HCC diagnostic factor (Figure 1d). Similar results were observed in another multistage liver disease dataset, GSE6764, further validating the increase in ITGA6 expression with advanced HCC stages and its remarkable diagnostic ability for HCC (Figure 1e) [12]. Additional analysis of nine publicly available HCC datasets confirmed that ITGA6 is significantly overexpressed in HCC in all datasets (Figure 1f).

Next, the correlation between ITGA6 expression and patient prognosis was explored. Utilizing the clinical information from the TCGA dataset, patient prognosis according to ITGA6 expression was assessed. Although no significant results were observed in overall survival, disease-free survival, or disease-specific survival in the entire patient data, a poorer prognosis was identified in patients with high ITGA6 expression in terms of progression-free survival (PFS) (Figure S1C and Figure 1g, left panel). Intriguingly, among 54 patients at stage G1, those with high ITGA6 expression showed a hazard ratio of 3.87, indicating a significantly higher risk (Figure 1g, right panel).

In summary, these results identify ITGA6 as a potential early diagnostic biomarker for HCC and reveal its prognostic implications.

### 3.2. Profiling ITGA6 Distribution in HCC’s Tumor Microenvironment

**Figure 2:**
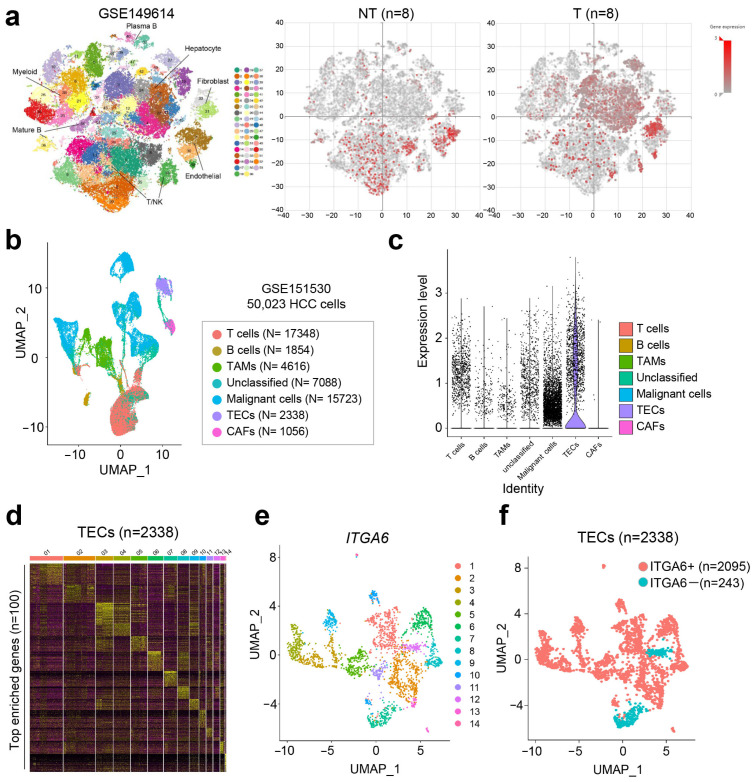
In-depth analysis of the ITGA6 expression in HCC tumor microenvironment. (**a**) The tSNE visualization of individual cell clusters from HCC patients (left). Comparison of ITGA6 expression profiles in normal liver and tumor tissues (middle and right). (**b**) UMAP of immune-associated and malignant cells colored by cell types analyzed with 50,023 cells from 25 HCC patients. TECs; tumor endothelial cells, CAFs; cancer-associated fibroblasts. (**c**) Violin plots of cell-type specific ITGA6 expression. (**d**) Visual representation of a heat map and clustering of the 100 most enriched genes in TECs (n = 2338). (**e**,**f**) UMAP plot of ITGA6 expression across the TECs colored by cell clusters.

To investigate the potential role of ITGA6 as a biomarker in the early diagnosis of HCC, scRNA-seq data of patient tissues from primary and metastatic tumors, GSE149614 was employed, and cell-specific expression levels in peritumoral non-cancerous and liver cancer tissues were measured [16]. Our t-distributed stochastic neighbor embedding (tSNE) plot analysis revealed a division into a total of 53 clusters (Figure 2a, left panel), and, of particular interest, we observed increased expression of ITGA6, notably within the hepatocytes of tumor tissues (Figure 2a, middle and right panels).

Even though the role of ITGA6 in HCC development is well established, the relation between ITGA6 level and immunity in the HCC microenvironment has not yet been elucidated. To assess the differences between ITGA6 expression levels across distinct immune cell populations, we obtained GSE151530, another scRNA-seq data set derived from HCC patient tissues [17]. We extracted 25 HCC patients from the sample set that comprises GSE151530 and performed UMAP analysis. The cells from HCC patients were grouped according to their lineage into malignant cells, T cells, B cells, tumor-associated macrophages (TAMs), cancer-associated fibroblasts (CAFs), and TECs, facilitated by the expression of lineage-specific marker genes (Figure 2b). Upon investigating the distribution of ITGA6 expression across different cells, it was intriguing to find that the expression of ITGA6 was particularly enriched in the TECs group (Figure 2c). A heatmap analysis was further conducted on the top 100 enriched genes within the TECs, leading to a classification into 13 clusters (Figure 2d). UMAP and violin plot analyses reaffirmed the widespread distribution of ITGA6 expression across most of these clusters (Figure 2e and Figure S2). Remarkably, out of the total 2338 TECs, 89.6% (2095 cells) exhibited positive ITGA6 expression (Figure 2f).

In summary, our results underline the potential of ITGA6 as an effective biomarker for the early diagnosis of HCC. These findings, derived from the in-depth analysis of two independent scRNA-seq datasets, underscore the importance of cell-specific biomarker discovery in cancer diagnosis and treatment.

### 3.3. Functional Significance of ITGA6-Positive TECs in HCC

The biological roles of the 606 genes identified in ITGA6-positive TECs were explored, utilizing gene ontology, and signaling pathway analysis to gain insights into the functional significance of these genes and their involvement in HCC progression.

**Figure 3:**
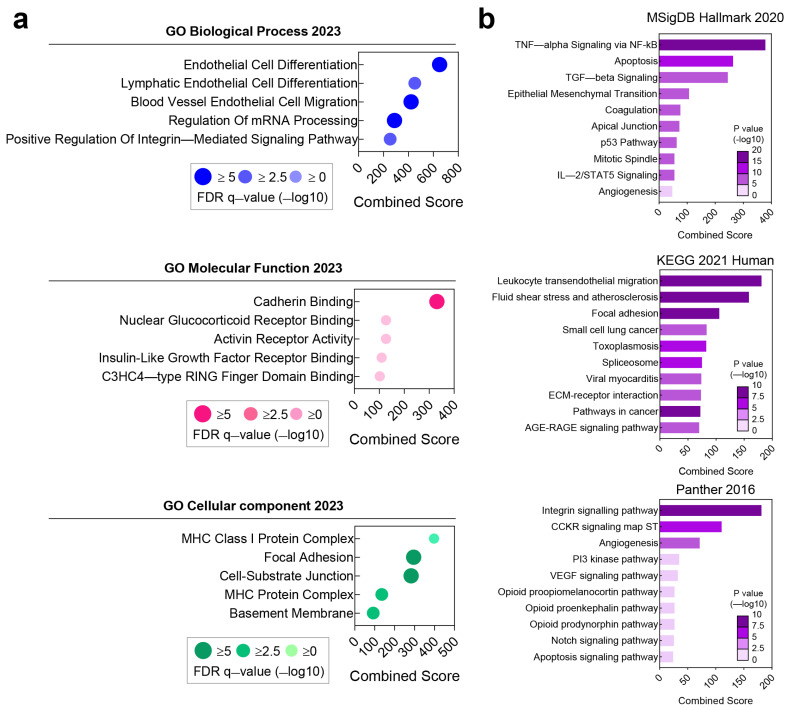
Predicting the function of ITGA6 in HCC TECs. (**a**) Top 5 gene ontology classifications of ITGA6 correlated genes in biological processes (top), molecular functions (middle), and cellular components (bottom). (**b**) Top 10 pathway analysis through GSEA with 606 ITGA6-associated genes in TECs.

The gene ontology analysis revealed that the most significantly enriched biological process associated with the ITGA6 co-expressed genes was endothelial cell differentiation (GO:0045446, combined score: 650.32, adjusted *p* = 4.73 × 10^−6^) (Figure 3a, top panel). This finding suggests that these genes play a crucial role in the differentiation of endothelial cells, which is essential for blood vessel formation. Furthermore, the molecular function analysis highlighted cadherin binding (GO:0045296, combined score: 331.13, adjusted *p* = 5.63 × 10^−20^) (Figure 3a, middle panel) as the most significantly associated function. Cadherins are cell adhesion molecules involved in cell–cell interactions and are known to play a role in endothelial cell differentiation. Regarding the cellular component analysis, the results emphasized the relevance of focal adhesion (GO:0005925, combined score: 295.39, adjusted *p* = 3.54 × 10^−20^) and cell-substrate junction (GO:0030055, combined score: 282.51, adjusted *p* = 4.87 × 10^−20^) (Figure 3a, bottom panel), both of which are components of the extracellular matrix and play crucial roles in cell adhesion and migration. These findings suggest that the co-expressed genes are involved in the interaction between endothelial cells and their surrounding environment, which is crucial for tumor angiogenesis and invasion.

Next, an analysis was conducted on 606 genes using the signaling pathway analysis with three databases—the MSigDB’s Hallmark (2020), KEGG pathway (2021), and Panther (2016). The analysis showed significant enrichments, such as TNF-alpha signaling via NF-kB (combined score: 378.63, adjusted *p* = 4.86 × 10^−19^) in the MSigDB Hallmark 2020 and leukocyte transendothelial migration (combined score: 180.94, adjusted *p* = 4.09 × 10^−9^) in the KEGG 2021 human pathway (Figure 3b, top and middle panels). Notably, the Panther 2016 pathway spotlighted the integrin signaling pathway (combined score: 181.35, adjusted *p* = 1.17 × 10^−10^) as the most enriched (Figure 3b, bottom panel).

These observations, in conjunction with the prominent emergence of critical pathways such as coagulation and angiogenesis, underscore the potential importance of ITGA6 and its co-expressed genes in TECs.

### 3.4. ITGA6 as an Immune Infiltration-Associated and Non-Invasive HCC Diagnostic Marker

To understand the interplay between ITGA6 and the immune landscape in HCC, further analysis was conducted on the correlation between ITGA6 expression and the infiltration levels of various immune cell types in the TCGA LIHC dataset using TIMER 2.0 (Tumor Immune Estimation Resource 2.0) [18,19,20].

**Figure 4:**
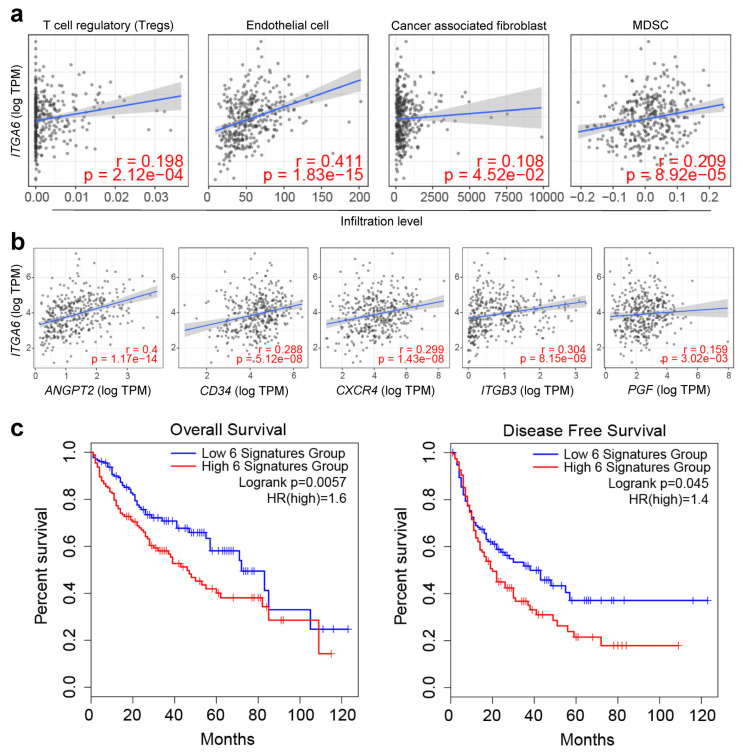
Validation of ITGA6 for immune-associated HCC diagnosis. (**a**) Immune cell infiltration analysis using TIMER 2.0 (http://timer.cistrome.org/, accessed on 23 March 2023). Correlation analysis of ITGA6 expression with the Tregs, TECs, CAFs, and MDSC cells in TCGA_LIHC. (**b**) Examining the correlation of ITGA6 expression with TECs markers. (**c**) Survival analysis with five markers related to ITGA6. Overall survival (left). Disease-free survival (right).

Consistent with our previous results, ITGA6 showed a significant positive correlation with endothelial cells (r = 0.411, *p* = 1.83 × 10^−15^). More intriguingly, ITGA6 also showed strong positive correlations with pro-tumorigenic immune cell types such as regulatory T cells (Tregs), CAFs, and myeloid-derived suppressor cells (MDSCs), highlighting a potential role in shaping a more permissive and supportive tumor microenvironment. (Figure 4a and Table S1). On the other hand, no notable correlations were found between ITGA6 expression and anti-tumorigenic immune cells, including CD8+ T cells, CD4+ T cells, and NK cells (Figure S3A). This suggests that ITGA6 may not be actively involved in recruiting or modulating these immune effectors, which are crucial for anti-tumor immunity.

To further explore the links between ITGA6 and established TECs markers, a correlation analysis was conducted with six known TECs marker genes [21,22,23]. Apart from ENG, a statistically significant positive correlation with ITGA6 was observed for the five marker genes ANGPT2, CD34, CXCR4, ITGB3, and PGF (Figure 4b and Figure S3B). The increased expression of ITGA6, along with its significant correlations with angiogenesis markers and immune cells, suggest that it might be involved in several key aspects of HCC biology. Moreover, survival analysis incorporating these five TEC markers and ITGA6 showed that higher expression of this six-gene signature is associated with poorer overall survival and disease-free survival, underscoring the clinical relevance of our findings (Figure 4c).

### 3.5. Diagnostic Relevance of ITGA6 in Liver Cancer

**Figure 5:**
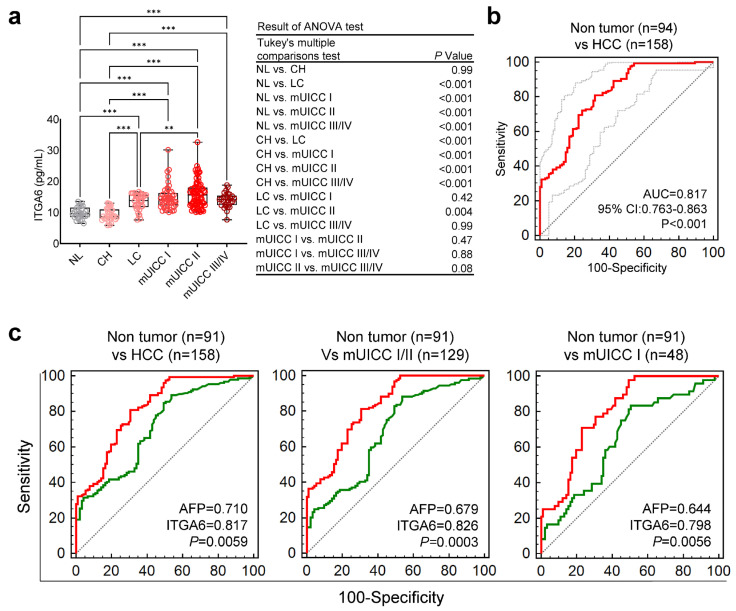
Concentration of serum ITGA6 protein and its diagnostic power in all-stage and early-stage HCC. (**a**) Box and point plot of serum ITGA6 expression, as measured by ELISA. Statistically significant differences were determined using the one-way ANOVA with Tukey’s multiple comparisons test. Black horizontal lines denote means, and error bars represent SEM. ** *p* < 0.01, *** *p* < 0.001 (**b**) Analysis of serum ITGA6 ROC curve in patients with HCC vs. control (NL, CH, and LC). Statistically significant differences in the AUC were relative to AUC of 0.5. (**c**) AUROCs for discriminating patients with all-stage HCC from the nontumor subjects (NL, CH, and LC) (left), AUROCs for discriminating patients with mUICC stage I or II HCC from nontumor subjects (healthy, CH, and LC) (middle) and AUROCs for discriminating patients with mUICC stage I tumors from the nontumor subjects (right). Statistically significant differences in AUC were between serum ITGA6 (red line) and serum AFP (green line).

To validate the potential of ITGA6 as a non-invasive diagnostic marker for HCC, the concentration of ITGA6 protein in serum was examined. Notably, ITGA6 protein concentration was significantly elevated in the patients with liver LC and HCC in comparison to those with NL and CH (** *p* < 0.01, *** *p* < 0.001, ANOVA-test) (Figure 5a).

Next, the potency of ITGA6 as a non-invasive biomarker was further confirmed through ROC analysis, with an AUC of 0.817, demonstrating robust diagnostic power for distinguishing non-tumor conditions from HCC (Figure 5b). Notably, when compared with the traditional HCC biomarker, AFP (green line), ITGA6 (red line) exhibited superior overall diagnostic performance (Figure 5c and Table 1).

These findings, taken together with previous results, underscore the potential of ITGA6 as a promising biomarker for early detection of HCC.

## 4. Discussion

HCC evolves through a progression that starts with DN formation due to liver tissue damage and transitions into early-stage and eventually advanced HCC [1,24]. Despite vigilant monitoring of high-risk patients, early-stage HCC presents a diagnostic challenge due to its subtle atypical features, making it difficult to differentiate between regenerative nodules, precancerous lesions, and actual early-stage HCCs [25]. There is an unmet need for non-invasive molecular markers that could standardize early-stage HCC diagnosis, guide treatment, and help recognize patients at high risk of postoperative recurrence.

In this context, our research emphasizes the significance of ITGA6, a gene encoding the α6 integrin subunit, in HCC, providing compelling evidence of a potential early diagnostic biomarker. Most notably, we found that ITGA6 overexpression presents consistently in early stage HCC patients, which provides evidence that ITGA6 overexpression occurs early in hepatocarcinogenesis, rather than simply being an outcome of advanced disease. Furthermore, we detected elevated concentrations of ITGA6 protein in the serum of HCC patients, demonstrating its potential as a non-invasive diagnostic marker.

Our investigation with scRNA-seq also revealed an intriguing increase in ITGA6 expression within hepatocytes and TECs in tumor tissues, pointing to a cell-specific expression pattern of ITGA6 in HCC. Genes enriched within ITGA6-positive TECs were related to endothelial cell differentiation and cell–cell interactions, suggesting a potential role for ITGA6 in angiogenesis.

Furthermore, over 90% of HCC cases are closely linked with inflammation and liver injury, underlining the crucial influence of the liver microenvironment in modulating disease progression [26,27]. Persistent inflammation causes an influx of immune cells into the liver, contributing to tissue remodeling [28]. It has been well documented that interactions between cancer cells and their surrounding TME play a pivotal role in cancer progression [29]. These interactions often result in the suppression of anti-cancer responses, as tumor cells adapt immune evasion strategies and reprogram immune cells to favor tumor growth [30]. As immune cells infiltrate, there is an imbalance in the production of chemokines and cytokines, coupled with an upsurge in the production of reactive oxygen species (ROS) and reactive nitrogen species. These changes contribute to fibrosis and cirrhosis, which can eventually lead to the malignant transformation of liver cells [31]. Moreover, in HCC, the role of IL-6 signaling via STAT3 was dual, based on the cell type releasing IL-6 and disease stage. The absence of IL-6 or STAT3 signaling in chronic liver inflammation enhanced HCC development, leading to an increase in hepatic steatosis, macrophage accumulation, and hepatocyte proliferation [32]. Tumor-associated neutrophils (TANs) regulate miR-301b-3p expression in stem-like HCC cells, influencing the NF-kB pathway and TAN infiltration, which modulate HCC progression [33,34]. Furthermore, the genetic backgrounds of HCC tumors, such as β-catenin activation, can affect their immunotherapy responses by altering cytokine expression and immune cell recruitment [35]. Additionally, research on the immune TME in the steatotic liver of Pten knockout mice suggests that macrophages might contribute to HCC development in steatotic TME [36]. MDSCs can enhance liver cancer cell stemness and impair dendritic cell function while suppressing T-cell infiltration. These cells can also foster MDSC expansion and immunosuppressive function via IL-6 secretion [37].

Hence, our findings suggest a potential role of ITGA6 in these processes, as it appears to positively correlate with pro-tumorigenic immune cells. This suggests that ITGA6 might contribute to the formation of a more immunosuppressive tumor microenvironment, favoring tumor growth and progression.

In addition, ITGA6 has a significant positive correlation with established markers of TECs, including ANGPT2, CD34, CXCR4, ITGB3, and PGF. This result indicates that ITGA6 could be involved in angiogenesis, a critical process in tumor growth and spread. The survival analysis also showed that a higher expression of a six-gene signature, including ITGA6 and the aforementioned five TEC markers, is associated with poorer overall and disease-free survival in HCC patients. This suggests that ITGA6 could serve as a potential prognostic marker in HCC.

Moreover, the serum concentration of ITGA6 protein was found to be significantly elevated in patients with liver LC and HCC, indicating its potential as a non-invasive diagnostic marker. The ROC analysis further confirmed this, demonstrating that ITGA6 has a strong diagnostic power for distinguishing HCC from non-tumor conditions, even exceeding the value of traditional biomarker, AFP. Taken together, our findings highlight ITGA6 as a potential early detection and prognostic biomarker for HCC. However, a comprehensive understanding of the role of ITGA6 in HCC necessitates further exploration, particularly into the molecular mechanisms underlying the enhanced expression of ITGA6 in HCC. One potential area for further research involves investigating the molecular pathways of ITGA6 in the onset of liver cancer. Understanding how ITGA6 interacts with other genes and molecules within these pathways could provide critical insights into the pathogenesis of HCC. Additionally, the relationship between ITGA6 and the highly immunosuppressive TME of HCC remains also unknown. Since TME plays a substantial role in the progression of HCC, uncovering the direct link between ITGA6 and TME could provide more precise targets for treatment and early detection strategies.

For a more comprehensive understanding, future research should delve into the mechanisms underlying the enhanced expression of ITGA6 in eHCC. Unlocking these molecular pathways could significantly refine early detection strategies, potentially innovating the therapeutic landscape of HCC.

## 5. Conclusions

Our study underscores the significance of ITGA6 in HCC, highlighting its potential as an early diagnostic and prognostic biomarker. Its consistent overexpression in eHCC and correlation with pro-tumorigenic immune cells suggest a key role in disease progression, prompting further investigation into its functional role and molecular mechanisms. Such research could ultimately contribute to refined detection strategies and improved clinical management of HCC.

## Figures and Tables

**Table 1:** The sensitivity and specificity of ITGA6 in detection of HCC.

Group	Criterion	Sensitivity	95% CI	Specificity	95% CI
Non tumor (n = 91) vs HCC (n = 158)	12.1917	81.01	74.0–86.8	69.23	58.7–78.5
Non tumor (n = 91) Vs mUICC I/II (n = 129)	12.1917	81.4	73.6–87.7	69.23	58.7–78.5
Non tumor (n = 91) vs mUICC I (n = 48)	10.3863	97.92	88.9–99.9	50.55	39.9–61.2

## Data Availability

RNA-sequencing datasets generated in this study and all other supporting data are available from the corresponding author upon reasonable request.

## Supplementary Materials

The following supporting information can be downloaded at: https://www.mdpi.com/article/10.3390/cancers15164156/s1, Figure S1: Analysis of ITGA6 expression levels across various tissues and cancer types. (A) GTEx Box-plot analysis of ITGA6 expression in various types of normal tissues. The green line represents the median expression of ITGA6 in liver tissues. (B) The differences in expression levels of ITGA6 mRNA in different tumors and normal tissues from TCGA. (C) The survival analysis of between the ITGA6_High and _Low groups. OS; overall survival, DFS; disease-free survival, DSS; disease-specific survival; Figure S2: Violin plot analysis of ITGA6 expression across the TECs numbered by cell clusters; Figure S3: Immune cell infiltration assessment based on ITGA6 expression using TIMER analysis. (A) The correlation analysis between ITGA6 and T cells with CD8, CD4 and NK cells. (B) The co-related expression analysis of the ENG gene with ITGA6. Table S1: Correlation of ITGA6 with Markers of Immune Cells in TCGA LIHC dataset.
