# Supplementary material for: Tumor Endothelial Cells-Associated Integrin Alpha-6 as a Promising Biomarker for Early Detection and Prognosis of Hepatocellular Carcinoma
Source: Cancers (Basel). 2023 Aug 17;15(16):4156. doi: 10.3390/cancers15164156 (PMC10453423; doi:10.3390/cancers15164156)
Supplement: Supplementary file 1 [file cancers-15-04156-s001.zip › cancers-2520879-supplementary.pdf]

Tumor Endothelial Cells-Associated Integrin Alpha-6 as a Promising Biomarker for Early Detection and Prognosis of Hepatocellular Carcinoma

Supplementary Materials

Table S1. Correlation of ITGA6 with Markers of Immune Cells in TCGA LIHC dataset

Description	Markers	ITGA6 (LIHC, n=371)	
		Corr	P value
T cell (general)	CD3D	0.021	6.96e-01
	CD3E	0.100	6.41e-02
	CD3G	0.27	3.69e-07
	CD2	0.065	2.26e-01
Cytotoxic T cell	CD8A	0.122	2.37e-02
	CD8B	0.03	5.75e-0.1
Regulatory T cell (Treg)	IL2RA	0.23	1.59e-05
	ENTPD1	0.451	1.07e-18
	FoxP3	0.212	7.23e-05
	CCR8	0.378	3.87e-13
Natural Killer (NK) cell	CD56 (NCAM1)	0.19	3.77e-04
	KLRD1	0.085	1.16e-01
	NKG2A (KLRC1)	-0.004	9.48e-01
	NKG2D (KLRK1)	0.011	8.37e-01
	NCR1 (NKp46)	0.178	9.28e-04
	NCR2 (NKp44)	0.064	2.38e-01
	NCR3 (NKp30)	-0.027	6.15e-01
Myeloid-Derived Suppressor Cells (MDSC)	HLA-DRA	0.222	3.05e-05
	HLA-DRB1	0.083	1.26e-01

	CD33	0.186	5.19e-04
	CD11b (ITGAM)	0.28	1.25e-07
	CD14 (negative)	-0.201	1.67e-04
	IDO(IDO1)	0.226	2.2e-05
	LOX	0.493	1.72e-22
	S100A8	0.032	5.52e-01
	S100A9	0.035	5.14e-01
	CD80	0.278	1.56e-07
	CD83	0.373	8.2e-13
B cell	CD45 (PTPRC)	0.410	1.87e-15
	CD19	0.051	3.49e-01
	CD79A	0.042	4.34e-01
Tumor Endothelial Cells (TECs)	CD34	0.288	5.12e-08
	ITGB3	0.304	8.15e-09
	ENG	0.028	6.05e-01
	CXCR4	0.299	1.43e-08
	PGF	0.159	3.02e-03
	ANGPT2	0.400	1.17e-14

Supplementary Figures

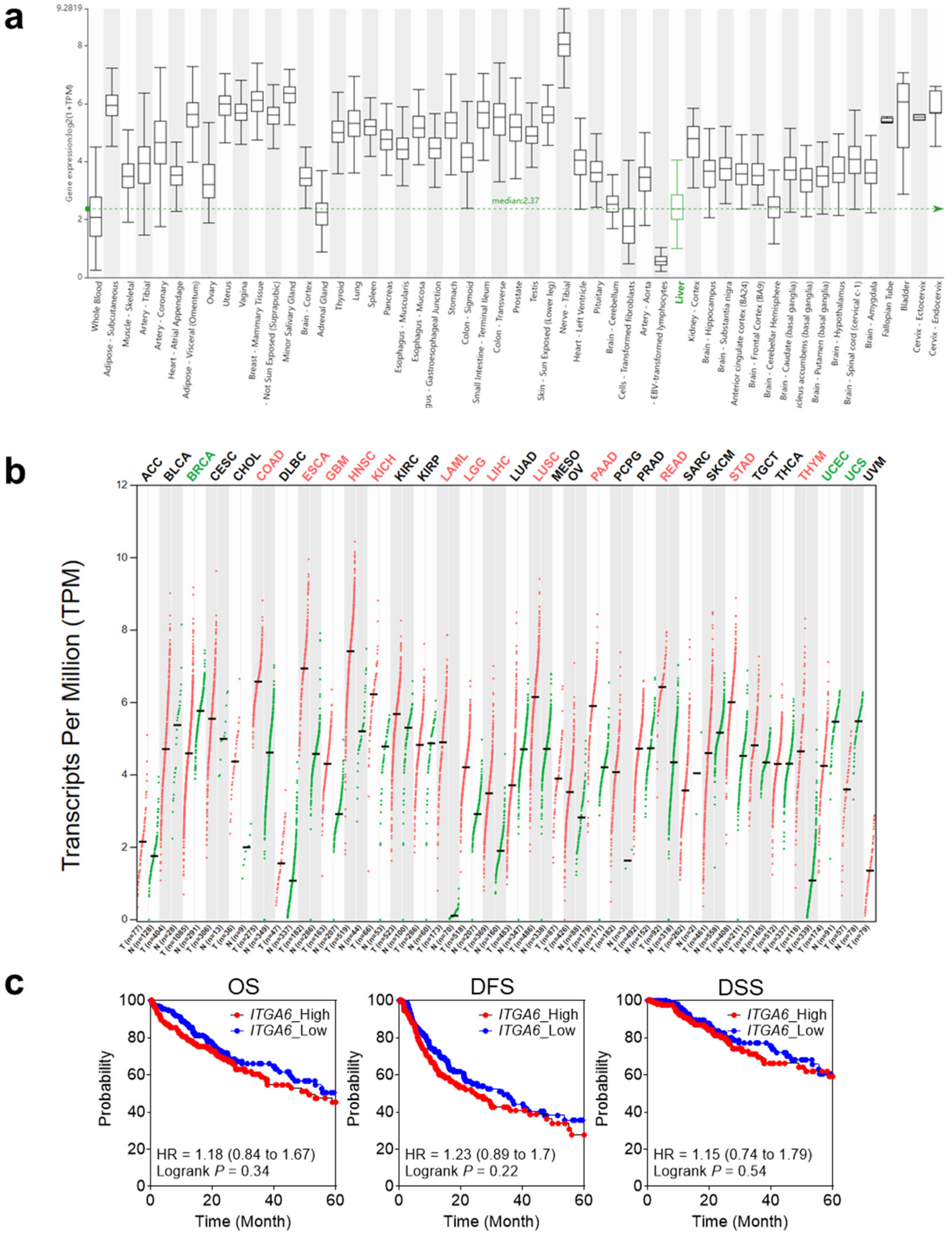

Figure S1 Analysis of ITGA6 expression levels across various tissues and cancer types. (A) GTEx Box-plot analysis of ITGA6 expression in various types of normal tissues. The green line represents the median expression of ITGA6 in liver tissues. (B) The differences in expression levels of ITGA6 mRNA in different tumors and normal tissues from TCGA. (C)

The survival analysis of between the ITGA6_High and _Low groups. OS; overall survival, DFS; disease-free survival, DSS; disease-specific survival.

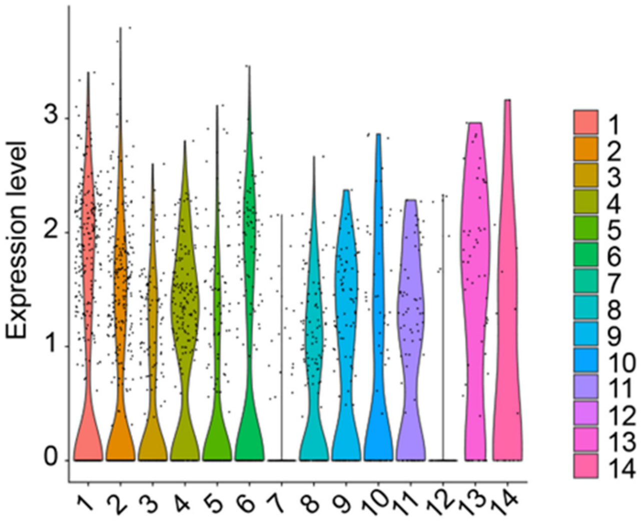

Figure S2 Violin plot analysis of ITGA6 expression across the TECs numbered by cell clusters.

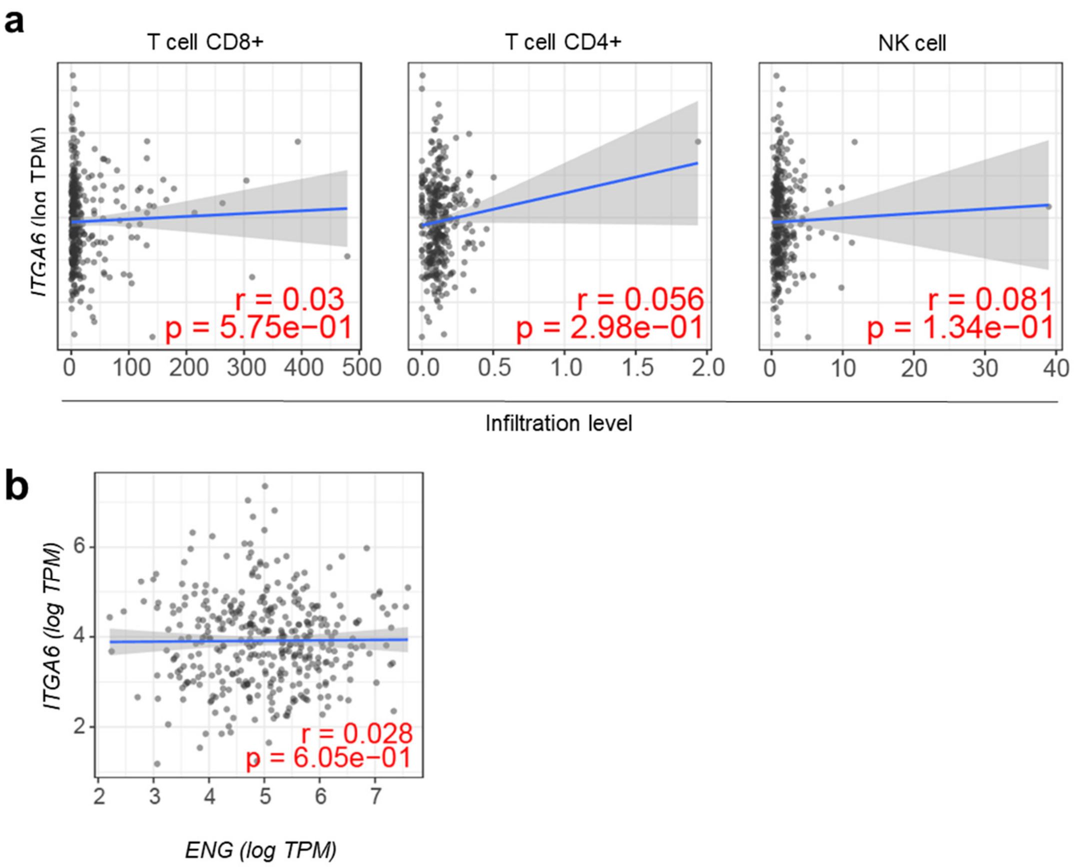

Figure S3 Immune cell infiltration assessment based on ITGA6 expression using TIMER analysis. (A) The correlation analysis between ITGA6 and T cells with CD8, CD4 and NK cells. (B) The co-related expression analysis of the ENG gene with ITGA6.
